# Extracellular Vesicles-Mediated Transfer of miRNA Let-7b from PC3 Cells to Macrophages

**DOI:** 10.3390/genes11121495

**Published:** 2020-12-12

**Authors:** Egidia Costanzi, Rita Romani, Paolo Scarpelli, Ilaria Bellezza

**Affiliations:** 1 Department of Medicine and Surgery, University of Perugia, 06123 Perugia, Italy; rita.romani@unipg.it (R.R.); ilaria.bellezza@unipg.it (I.B.); 2 Faculty of Psycology, Università degli Studi eCampus, 22060 Novedrate, Italy; paolo.scarpelli@uniecampus.it

**Keywords:** prostate cancer, exosomes, microRNA, tumor-associated macrophages

## Abstract

Prostate-derived extracellular vesicles (pEVs) may represent a way to selectively transport cargo molecules from the producing cells to the target cells to allow biological events, both in physiological and pathological circumstances. pEVs cargo participates in the modulation of the inflammatory responses in physiological conditions and during cancer progression. In the present study, we examined the expression levels of miRNA Let-7b, in both precursor and mature forms, in noncancerous and cancerous prostate cell lines, PNT2 and PC3 respectively, and in their extracellular vesicles (EVs) using reverse-transcription quantitative PCR strategies. We showed that miRNA Let-7b was highly expressed in noncancerous cells and strongly decreased in cancerous PC3 cells, while the opposite was observed in the respective EVs, thus supporting the tumor suppressor role of miRNA Let7-b. We also demonstrated that miRNA Let-7b can be transferred to THP-1 cells via EVs, which are known to induce TAM-like polarization. Our results support the view that miRNA Let-7 b, contained in PC3-derived EVs, is associated with the increase in the miRNA Let7-b observed in TAM-like macrophages. Overall, our results indicate that circulating EV-loaded miRNA might be useful biomarkers for prostate cancer progression and might also support a possible use of pEVs as targets for prostate cancer therapy.

## 1. Introduction

Prostate cancer is one of the main causes of cancer and the fifth leading cause of cancer death in men worldwide [1]. Many different cell types such as epithelial cells, fibroblasts, endothelial cells and immune cells contribute to prostate gland composition [2], and many of them are involved in prostate tumor onset and progression. The prostate gland is devoted to the synthesis and secretion of proteins and enzymes into the seminal fluid. Such molecules regulate specific physiological functions including motility of sperm cells, acrosome reaction and immune suppression within the female reproductive tract [3]. Moreover, prostatic fluid contains extracellular vesicle (EVs), historically known as prostasomes, which are released into the lumen of the prostatic ductal system from the epithelial cells of the gland [4]. A growing body of evidence indicates that cell-cell communication between cancer cells and other cells of the tumor microenvironment is pivotal for cancer progression [5,6,7]. Among the communication systems, prostate-derived extracellular vesicles (pEVs) have received particular attention for their involvement in the initiation, progression and metastasis of prostate cancer [8]. pEVs are surrounded by a lipid bilayer and contain membrane and soluble proteins, mRNAs, miRNAs and single- and double-stranded DNA molecules. It has been demonstrated that a subset of miRNA is preferentially loaded into EVs [9] and that EVs contain a higher number of specific miRNAs compared to their parent cells [10]. Regulation of gene expression by miRNA molecules is a conventional function of these biomolecules; for example, miRNAs exert a modulatory effect of several inflammatory signaling pathways [11]. To this respect, aberrant miRNA Let-7 expression has been associated with a variety of human diseases such as cardiovascular diseases, cystic fibrosis, metabolic disease as type 2 diabetes mellitus and cancer [12,13,14,15,16]. The Let-7 gene encodes a highly conserved miRNA family, whose members have generally thought to be overlapping because of sequence similarity [15,16]. The Let-7 miRNA family exerts tumor suppressor activities by targeting several oncogenes implicated in cell-cycle progression, cell proliferation, migration, differentiation and epithelial-to-mesenchymal transition (EMT) [15,16]. Therefore, the acquisition of oncogenic mutations and the downregulation of miRNA Let-7 might represent critical early steps in the oncogenic transition [16,17]. In inflamed tissue, however, a distinct expression pattern of miRNA Let-7 b compared with healthy controls was observed and was related to the increase in prostatic tumor-associated macrophages (TAMs), whose presence is linked to enhanced tumor progression [4]. Since we recently demonstrated that PC3-derived EVs induce a M2/TAM-like phenotype in THP-1 monocytic cells [6], we aimed at determining whether miRNA Let-7b could be loaded onto PC3-derived EVs and whether it could be transferred to THP-1 cells. We reported the analysis of expression levels of miRNA Let-7b in a noncancerous prostatic PNT2 cell line and a castration-resistant prostate cancer PC3 cell line and in the EVs they secrete. We observed a higher miRNAs Let-7b content in PC3-derived pEVs, suggesting their selective loading in cancer derived EVs as a part of the molecular signaling leading to cancer initiation/progression. 

## 2. Materials and Methods 

### 2.1. Material

All the reagents, unless otherwise stated, were from Merck KGaA (Darmstadt, Germany). Cell culture reagents were from Life Technologies (GibcoBRL, Gaithersburg, MD, USA).

### 2.2. Cell Culture and Treatments

Human androgen-independent prostate cancer PC3 cells, human noncancerous prostate epithelial PNT2 cells and the monocytic THP-1 cell line were obtained from the American Type Culture Collection (ATCC, Manassas, VA, USA). Cell lines were maintained at 37 °C in 5% CO_2_ in RPMI 1640 supplemented with 10% heat-inactivated FBS, 1× l-glutamine, 100 units/mL of penicillin and 0.1 mg/mL of streptomycin (Invitrogen, Monza, Italy). Human PBMCs were obtained from whole blood of voluntary healthy donors and isolated by a conventional hystopaque gradient. Prostate cell lines were grown until confluence, changing medium if necessary. THP-1 cells were seeded at 5 × 105 in 2 mL of medium in 6-well plates and exposed to 300 nM 12-O-tetradecanoilforbol-13-acetato (TPA). Three days later the cells were exposed to 100 μg/mL of PC3-derived extracellular vesicles (PC3-EVs) or to conditioned medium deprived of extracellular vesicles (PC3 CM-EVs), or they were treated with 20 ng/mL human IL-4 for 1 h. 

### 2.3. Isolation of Extracellular Vesicles (EVs) and Preparation of Conditioned Medium Depleted of EVs (CM-EVs)

Extracellular vesicles were isolated as previously described [6]. Briefly, PC3 cells were grown in RPMI 1640 supplemented with extracellular-vesicles-depleted FBS for 3 days. Cell culture medium was pooled and subjected to differential centrifugation. The resulting supernatant was ultracentrifuged at 100,000× g for 2 h in an Optima TLX ultracentrifuge with a 60 Ti rotor (Beckman Coulter, Brea, CA, USA) to recover the supernatant, consisting in a conditioned medium deprived of extracellular vesicles (CM-EVs) and pellets containing extracellular vesicles (EVs). The EV pellets were resuspended in PBS supplemented with 1% penicillin/streptomycin. Protein concentration was evaluated by measuring absorbance at 280 nm assuming *ε*_M_ = 1. CM-EV and EV preparations were stored at −80 °C until use.

### 2.4. Extracellular Vesicles Staining and Fluorescence Microscopy

Extracellular vesicles were stained with 50 μM 1,1′-dioctadecyl-3,3,3′,3′-tetramethylindodicarbocyanine,4-chlorobenzenesulfonate salt (DiD’; Thermofisher, Carlsbad, CA, USA) for 30 min, as previously described [6]. TPA-differentiated THP-1 cells, seeded on glass coverslips, were exposed to 100 μg/mL of DiD’-stained PC3-EVs. At the indicated time points, cells were fixed with 4% PFA for 20 min at room temperature, and F-actin was stained with fluorescein isothiocyanate (FITC)-labeled phalloidin (1:250) for 30 min at room temperature. Cell nuclei were counterstained with 4′,6′-diamidino-2phenylindole (DAPI). Cells were then rinsed in PBS, mounted and analyzed with a Zeiss Axio Observer Z1 equipped with Apotome and digital Camera Axiocam MRm (Zeiss, Oberkochen, Germany).

### 2.5. RNA Isolation, cDNA Synthesis, PCR and QPCR

Total RNA was extracted from PNT2, PC3 or THP-1 cells and PNT- or PC3-derived EVs using TRI reagent (Sigma-Aldrich, Milan, Italy) according to the manufacturer’s instructions. cDNA was synthesized from 1 μg total RNA using PrimeScript TM RT Reagent Kit (Takara Bio Europe, Saint-Germain-en-Laye, France). Reverse transcription for pre-miRNA molecules was performed using random oligonucleotide mix as primer to avoid exclusive mRNAs recovery. First-strand cDNA synthesis for miRNA was performed using a specific stem loop primer to selectively transcribe mature miRNA molecules. qPCR was carried out using SYBR Green Jump Start ready mix (Sigma-Aldrich, Milan, Italy), according to the manufacturer’s instructions, in a 25 μL of reaction volume in the presence of 40 ng of cDNA and 400 nM primer sets specific for pre-miRNA and miRNA targets. U6 snRNA was used as a housekeeping reference. In each assay, no-template controls were included, and each sample was run in triplicate. The thermal profile consisted of incubation at 95 °C for 10 min, followed by 40 cycles of denaturation at 95 °C for 30 s, annealing at 58 °C for 30 s and extension at 72 °C for 20 s. Mean *Ct* values of the samples were compared to the untreated control sample, and U6 was used as internal control. The n-fold differential ratio increase was expressed as 2^−ΔΔCt^. PCR primer sequences, obtained from Invitrogen (Invitrogen Ltd., Paisley, UK), are listed in Table 1. Pre- and miRNA PCR amplification were performed from cDNA isolated from PC3 and PNT2 cells and total EVs or EV fractions and visualized on 2% ethidium bromide-stained high-resolution agarose gel (Sigma-Aldrich, Milan, Italy). Two microliters of cDNA were used as a target for each PCR reaction using Taq DNA polymerase (New England Biolabs, Whitby, ON, Canada), according to the manufacturer’s protocol. A forward nested primer was designed internal to the pre-miRNA sequence. 

### 2.6. Statistical Analysis

All results were confirmed in at least three separate experiments and expressed as mean ± S.D. Data were analyzed for statistical significance by Student’s *t*-test. *p*-values < 0.05 were considered significant.

## 3. Results

### 3.1. Expression of Endogenous miRNA Let-7b in Cancerous and Noncancerous Prostate Cell Lines

**Figure 1:**
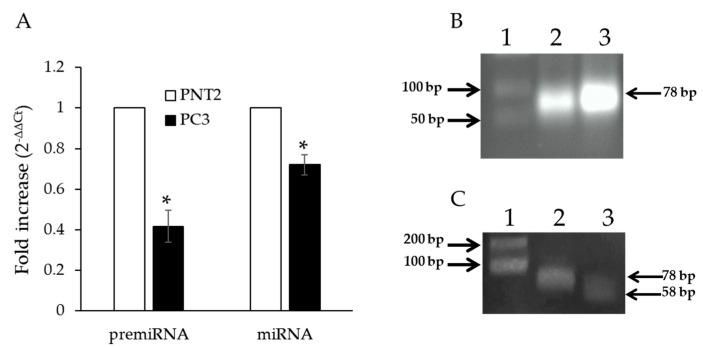
Endogenous miRNAs Let-7b in PC3 and PNT2 cell lines. (**A**) qPCR of pre-miRNA Let-7b and mature miRNA Let-7b in PC3 and PNT2 cells. Gene expression values were normalized to U6 snRNA and presented as 2^−ΔΔCt^. Relative RNA abundance in PNT2 cells was assumed as 1. Data represent mean ± SD (*n* = 5). * *p* < 0.05. (**B**) Semiquantitative PCR pre-miRNA Let-7b in PC3 (lane 2) and PNT2 cells (lane 3). (**C**) PCR pre-miRNA Let-7b (lane 2) and heminested pre-miRNA Let-7b (lane 3) in PC3 cells.

miRNA molecules represent key participants in the regulation of gene expression and play essential roles in the development of oncological diseases, including prostate cancer [18]. On the basis of these premises, we analyzed the content of both precursor and mature miRNA Let7-b in PC3 and PNT2 cells since they represent in vitro models for advanced-stage prostate cancer and noncancerous prostate epithelium. We found a 60% lower level of pre-miRNA Let-7b and a 30% lower level of mature miRNA Let-7b in PC3 cells compared to noncancerous PNT2 cells (Figure 1A). Our data correlated with the previously reported observation that miRNA Let-7 is downregulated in prostate cancer [17,19]. 

The differential expression of pre-miRNA Let-7b in the two cell lines can also be observed in the 2% agarose gel where the amplicons from semiquantitative PCR were loaded (Figure 1B). To verify that the amplified product corresponded to pre-miRNA Let-7b, we performed heminested PCR with a specific internal primer. As shown in Figure 1C, the smaller size of the heminested PCR amplicon (58 bp) confirmed the correctness of the amplified target. Our results showing that miRNA Let-7b is differentially expressed in prostate tumor cells serve to strengthen the hypothesis that it could act as a tumor suppressor gene [20]. 

Given that prostate cells are highly involved in the active secretion of EVs, we hypothesized that miRNAs could also be specifically loaded into pEVs and perform its functions on target cells by EV-mediated cargo transfer.

### 3.2. Differential Let-7b miRNA Content in EVs Compared to Prostate Cells

On the basis of the concept that the specific molecules loaded on EVs may not be in the same proportion as they are in the cell from which they originate [10], we analyzed the possible difference in the pre-miRNA Let-7b and mature miRNA Let-7b loaded in EVs compared to that of the prostate cell lines from which they derive. 

**Figure 2:**
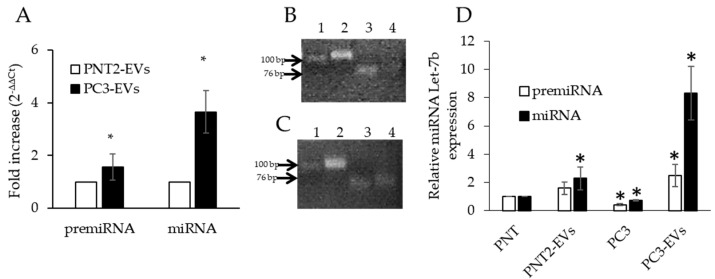
miRNAs Let-7b in EVs. (**A**) qPCR of pre-miRNA Let-7b and mature miRNA Let-7b in PNT2-derived EVs (PNT2-EVs) and PC3-derived EVs (PC3-EVs). Gene expression values were normalized to U6 snRNA and presented as 2^−ΔΔCt^. Relative RNA abundance in PNT2-EVs was assumed as 1. Data represent mean ± SD (*n* = 4). * *p* < 0.05 vs. PNT2-EVs. (**B**) Mature miRNA Let-7b (lane 3) and pre-miRNA Let-7b (lane 4) in PNT2-EVs, normalized for U6 (lane 2). (**C**) Mature miRNA Let-7b (lane 3) and pre-miRNA Let-7b (lane 4) in PC3–EVs, normalized for U6 (lane 2). (**D**) Comparative analysis of the expression levels of precursor and mature forms of prostate cells and their pEVs. Data represent mean ± SD (*n* = 4). * *p* < 0.05 vs. PNT2.

PC3-derived EVs (PC3-EVs) contain one and a half times higher pre-miRNA Let-7b than PNT2-derived EVs (PNT2-EVs), whereas the mature miRNA Let-7b was almost four times higher in PC3-EVs than in PNT2-EVs (Figure 2A). Figure 2B,C shows the respective PCR amplicons loaded on 2% agarose gel.

To better evaluate the relative amount of the precursor and mature forms of miRNA Let-7b in prostate cells and the respective EVs, we charted the expression levels of pre-miRNA Let-7b and mature miRNA Let-7b, assuming PNT2 cells as control, and set them as one for both molecules. As shown in Figure 2D, PNT2-EVs were slightly enriched in pre- and mature-miRNA Let-7b, thus suggesting that miRNA Let-7b has the tendency to be secreted. On the other hand, we observed that PC3-EVs were highly enriched in the precursor form and even more in the mature form of miRNA Let7-b compared to noncancerous PNT2 cells or PNT2-EVs or PC3 cells themselves. These data indicate that both the precursor and the mature miRNA Let-7b are strongly increased in PC3-derived EVs.

These results also highlight that PC3-cells have the greatest tendency to load miRNA Let-7b, especially in the mature form, into EVs in order to secrete it. The mechanism and the molecules involved in this process still need to be clarified. 

### 3.3. Evaluation of miRNA Let-7b in THP-1 Cells Treated with PC3-Derived EVs

**Figure 3:**
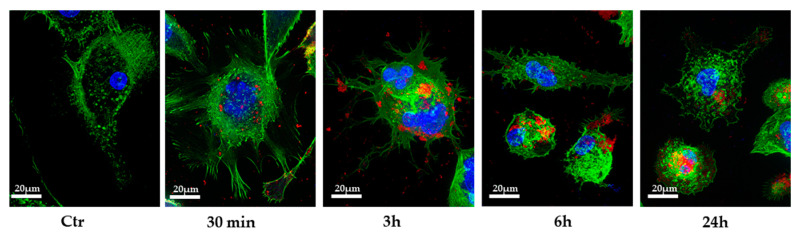
Intracellular localization of EVs. TPA-differentiated THP-1 cells were exposed to 100 μg/mL DiD’-stained PC3-EVs. At the indicated time points, cells were fixed, actin filaments were stained with FITC-labeled phalloidin and nuclei were counterstained with DAPI. The images are representative of one out of three separate experiments. Magnification 63×.

Several studies reported that miRNAs could be transferred between cells via EVs [6,7,21]; thus, we wondered whether PC3-derived EVs could transfer miRNA Let-7b to THP-1 cells. We exposed THP-1 monocytic cells, after TPA differentiation into M0 macrophages, to 100 μg/mL PC3-EVs for different times and observed the appearance of red fluorescent EV-like particles in the cytoplasm after 30 min incubation (Figure 3. These data suggest that THP-1 cells rapidly uptake PC3-derived EVs. This highlights that EV-like particles remain detectable inside the cells even after 24 h incubation. However, because EVs are metabolized into the multivesicular bodies upon internalization, the labeling of this intracellular compartment by dye diffusion might not be ruled out.

**Figure 4:**
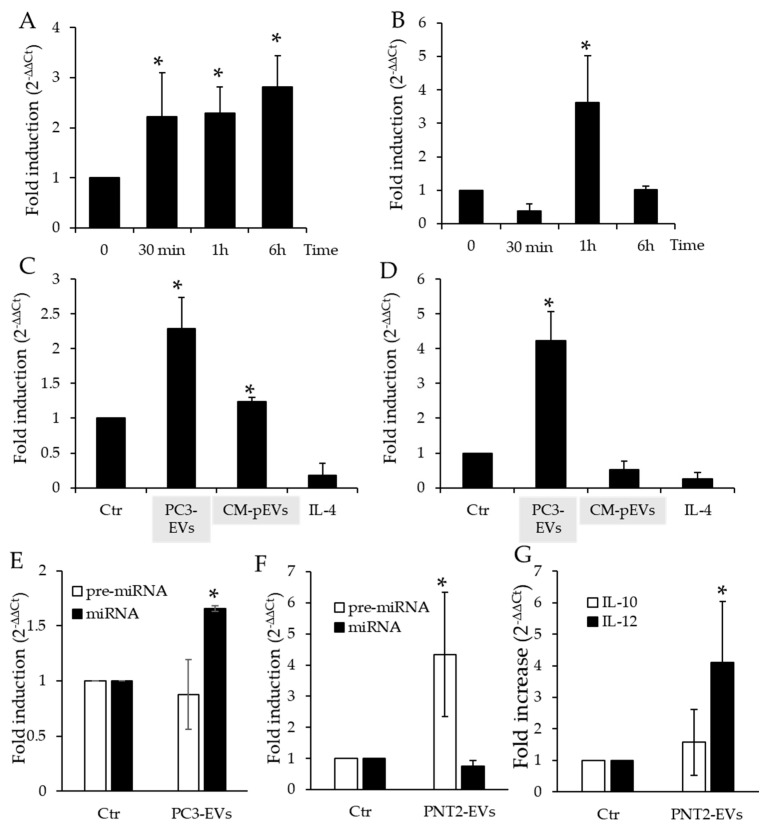
Evaluation of miRNAs Let-7b in THP-1 treated with PC3-derived EVs (PC3-EVs). (**A**) qPCR of pre-miRNA Let-7b in THP-1 cells treated for the indicated time points with PC3-EVs. (**B**) qPCR of mature miRNA Let-7b in THP-1 cells treated for the indicated time points with PC3-EVs. (**C**) qPCR pre-miRNA Let-7b in differentiated THP-1 cells after a 1 h treatment with PC3-EVs, CM-pEVs or IL-4. (**D**) qPCR of mature miRNA Let-7b in differentiated THP-1 cells after 1 h treatment with PC3-EVs, CM-pEVs or IL-4. (**E**) qPCR of pre-miRNA Let-7b and miRNA Let-7b in PBMC cells treated for 1 h with PC3-EVs. (**F**) qPCR of pre-miRNA Let-7b and miRNA Let-7b in THP-1 cells treated for 1 h with PNT2-EVs. (**G**) qPCR of IL-12 and IL-10 in THP-1 cells treated for 6 h with PNT2-EVs. Gene expression values were normalized to HPRT and presented as 2^−ΔΔCt^. Relative mRNA gene abundance in untreated cells (Ctr) was assumed as 1. Data represent mean ± SD (*n* = 4). * *p* < 0.05.

In order to determine whether EV uptake results in an increase in pre-miRNA Let-7b and mature miRNA Let-7b in THP-1 cells, we performed qPCR of pre-miRNA Let7b and mature miRNA Let-7b in THP-1 treated with PC3-derived EVs from 30 min up to 6 h. We found that the pre-miRNA Let-7b levels increased after 30 min exposure and were maintained throughout the course of the experiment. On the other hand, the levels of mature miRNA Let-7b strongly increased after a one-hour incubation and returned to control levels after 6 h (Figure 4A,B).

In addition to correlating with the finding that exposure to PC3-conditioned medium induces TAM-like polarization in THP-1 cells by increasing intracellular miRNA Let-7b expression [22], these data suggest that the increase in the content of miRNA Let-7b observed in THP-1 cells depends on EV-mediated miRNA transfer and not an increase in miRNA expression by THP-1 cells themselves. To verify this hypothesis, we exposed TPA-differentiated THP-1 cells to PC3-conditioned medium deprived of EVs (CM-pEVs) and to IL-4, a classical inducer of M2 differentiation, for 1 h and determined intracellular miRNA Let-7b expression (Figure 4C,D). We found that exposure of THP-1 cells to CM-pEVs caused a small yet significant increase in the expression of pre-miRNA Let-7b, whereas exposure to IL-4 reduced pre-miRNA Let-7 b expression (Figure 4C). In the same experimental conditions, the expression of mature miRNA Let-7b was not affected by exposure to either CM-pEVs or IL-4 (Figure 4D). These results demonstrated that the prompt increase in miRNA Let-7b in THP-1 cells was due to PC3-derived EVs uptaken by monocytic cells. We also exposed PBMCs isolated from human blood to PC3-EVs and found that they induced a mild increase in miRNA Let-7b while leaving the precursor level unaltered (Figure 4E).

We also incubated THP-1 cells with PNT2-derived EVs and found that a 1 h incubation with PNT2-EVs did not affect miRNA Let-7b and only modestly increased pre-miRNA Let-7b intracellular levels (Figure 4F). Moreover, we also found that 6 h exposure to PNT2-EVs did not induce M2-like polarization in THP-1 cells, as evidenced by the gene expression profile (Figure 4G). Hence, while PC3-EVs treated THP-1 are characterized by a IL-12^low^/IL-10^high^ profile [6], THP-1 cells exposed to PNT2-EVs only display a modest increase in IL-12 gene expression.

Overall, these results demonstrate that miRNA Let-7b is transported by EVs from PC3 cells to monocytic cells where it may participate in TAM polarization.

## 4. Discussion

In this study, we characterized miRNA Let-7b distribution in prostate noncancerous and cancerous cell lines and in the EVs they produce. In the healthy prostate, immune-suppressive activity exerted by EVs represents a physiological function crucial for the fertilization process [3,23,24]. However, this important physiological feature can also be exploited by tumor cells. In fact, it is known that the immune suppression properties of the EVs can favor tumor progression, as they might affect macrophage polarization participating in the tumor immune escape [11]. Tumor-derived EVs are indeed capable of inhibiting the immune system functions either by direct internalization and alteration of the target immune cells or through the interaction and resetting of stromal cells, which, in turn, can affect the immune cell behavior. Independently of the mechanism, EVs seem to induce a TAM-like phenotype in peritumoral macrophages [6,25]. To this respect, recent observations reported miRNA molecules as active participants in this process [26]. For example, Valadi and collaborators demonstrated the exosome-mediated transfer of different types of RNA, including miRNAs, between cells [27]. We found that pre-miRNA Let-7b and mature miRNA Let-7b are loaded in EVs isolated from both noncancerous PNT2 and cancerous PC3 prostate cell lines. Nonetheless, a differential miRNA export can be inferred by the finding that PC3-derived EVs contain a four-times higher level of miRNA Let-7b compared to PNT2 cells. These differences in miRNA distribution may be the consequence of different expression levels or of a higher loading rate in PC3-derived EVs. The finding that prostate-cancer-derived PC3 cell lines express a lower level of miRNA Let-7b does not support the first hypothesis. On the other hand, the second scenario might be the best biological solution because tumor cells, by decreasing the intracellular levels of the tumor suppressor miRNA Let-7b, may increase cell proliferation and contextually secrete a molecule capable of supporting an immunosuppressive tumor-promoting microenvironment. 

We have previously shown that PC3-derived EVs induce a TAM-like phenotype in THP-1 cells [6], and here we showed that EVs isolated from noncancerous PNT2 cells did not exert the same effects. Banerjee and co-workers found that the miRNA Let-7c regulated M0 macrophages polarization in a murine cellular model [28], and Wang and colleagues showed that PC3-conditioned medium induced the polarization of THP-1 cells into TAMs (tumor-associated macrophages) by the upregulation of miRNA Let-7b [22]. Here, we found that 1 h exposure to EVs is sufficient to increase the levels of miRNA Let-7b in differentiated THP-1 cells and human PBMCs. Although the role of miRNA Let-7b in the macrophage polarization process was already demonstrated [22], we showed, for the first time, that in order to exert its biological function, miRNA Let-7b must be loaded in EVs rather than secreted as a soluble molecule in the extracellular space. These results might be explained by the absence of the pre-miRNA maturating RNAse type III (Dicer) in THP-1 cells [29].

Future studies focusing on the molecular pathways leading to selective miRNA loading into EVs are needed. Moreover, to define whether the amount of miRNA Let-7b loading on EVs correlates with the stages of tumor progression, production and EVs loading of miRNA Let-7b by other prostate cancer cell lines together with the analysis of its immune effects needs to be further investigated.

## 5. Conclusions

Overall, these results demonstrate that PC3-derived EVs contain important signaling molecules, such as miRNA Let-7b, involved in the generation of a tumor-promoting microenvironment and suggest their potential use as targets for cancer therapy [26]. Moreover, the urgent need for new biomarkers for diagnosis and risk-stratification in prostate cancer [30,31,32] might lead to the use of EV-loaded miRNA Let-7b as a candidate biomarker for tumor progression. 

## Figures and Tables

**Table 1:** List of Primers.

Gene	Forward (F)	Reverse (R)
Pre-miRNA Let-7b	5′ TGAGGTAGTAGGTTGTGTGG 3′	5′ CAGGGAAGGCAGTAGGTTG 3′
nested pre-miRNA Let-7b	5′ TTTCAGGGCAGTGATGTTGC 3′	
miRNA Let-7b	5′ TGAGGTAGTAGGTTGTGTGGTT 3′	5′ GTGCAGGGTCCGAGGT 3′
IL-12 p40	5′ CGGTCATCTGCCGCAAA 3′:	5′ TGCCCATTCGCTCCAAGA 3′
IL-10	5′ CGAGATGCCTTCAGCAGAGT 3′	5′ CGCCTTGATGTCTGGGTCTT 3′
stem loop miRNA Let-7b	5′ GTCGTATCCAGTGCAGGGTCCGAGGTGCACTGGATACGACCACCCACCAACCAC 3′	

## References

[1] Bray F., Ferlay J., Soerjomataram I., Siegel R.L., Torre L.A., Jemal A. (2018). Global cancer statistics 2018: GLOBOCAN estimates of incidence and mortality worldwide for 36 cancers in 185 countries. CA Cancer J. Clin..

[2] Barron D.A., Rowley D.R. (2012). The reactive stroma microenvironment and prostate cancer progression. Endocr. Relat. Cancer.

[3] Kelly R.W. (1995). Immunosuppressive mechanisms in semen: Implications for contraception. Hum. Reprod..

[4] Raposo G., Stoorvogel W. (2013). Extracellular vesicles: Exosomes, microvesicles, and friends. J. Cell Biol..

[5] Niazi V., Parseh B., Ahani M., Karami F., Gilanchi S., Atarodi K., Soufi M., Soleimani M., Ghafouri-Fard S., Taheri M. (2020). Communication between stromal and hematopoietic stem cell by exosomes in normal and malignant bone marrow niche. Biomed. Pharmacother..

[6] Mezzasoma L., Costanzi E., Scarpelli P., Talesa V.N., Bellezza I. (2019). Extracellular vesicles from human advanced-stage prostate cancer cells modify the inflammatory response of microenvironment-residing cells. Cancers.

[7] Lucidi A., Buca D., Ronsini C., Tinari S., Bologna G., Buca D., Leombroni M., Liberati M., D’Antonio F., Scambia G. (2020). Role of extracellular vesicles in epithelial ovarian cancer: A systematic review. Int. J. Mol. Sci..

[8] Ronquist G. (2012). Prostasomes are mediators of intercellular communication: From basic research to clinical implications. J. Intern. Med..

[9] Guduric-Fuchs J., O’Connor A., Camp B., O’Neill C.L., Medina R.J., Simpson D.A. (2012). Selective extracellular vescicle-mediated export of an overlapping set of microRNAs from multiple cell types. BMC Genom..

[10] Goldie B.J., Dun M.D., Lin M., Smith N.D., Verrills N.M., Dayas C.V., Cairns M.J. (2014). Activity-associated miRNA are packaged in Map 1b-enriched exosomes released from depolarized neurons. Nucleic Acid Res..

[11] Greening D.W., Gopal S.K., Xu R., Simpson R.J., Chen W. (2015). Exosomes and their roles in immune regulation and cancer. Semin. Cell Dev. Biol..

[12] Bao M.H., Feng X., Zhang Y.W., Lou X.Y., Cheng Y., Zhou H.H. (2013). Let-7 in cardiovascular diseases, heart development and cardiovascular differentiation from stem cells. Int. J. Mol. Sci..

[13] Ideozu J.E., Zhang X., Rangaraj V., McColley S., Levy H. (2019). Microarray profiling identifies extracellular circulating miRNAs dysregulated in cystic fibrosis. Sci. Rep..

[14] López-Pastor A.R., Infante-Menéndez J., Escribano Ó., Gómez-Hernández A. (2020). miRNA dysregulation in the development of non-alcoholic fatty liver disease and the related disorders type 2 diabetes. Front. Med..

[15] Wang X., Cao L., Wang Y., Wang X., Liu N., You Y. (2012). Regulation of Let-7 and its target oncogenes (Review). Oncol. Lett..

[16] Wagner S., Ngezahayo A., Murua Escobar H., Nolte I. (2014). Role of miRNA Let-7 and its major targets in prostate cancer. Biomed. Res. Int..

[17] Iliopoulos D., Hirsch H.A., Struhl K. (2009). An epigenetic switch involving NF-κB, Lin28, Let-7MicroRNA, and IL6 links inflammation to cell transformation. Cell.

[18] Konoshenko M.Y., Bryzgunova O.E., Laktionov P.P. (2020). miRNAs and radiotherapy response in prostate cancer. Andrology.

[19] Nadiminty N., Tummala R., Lou W., Zhu Y., Shi X.B., Zou J.X., Chen H., Zhang J., Chen X., Luo J. (2012). MicroRNA Let-7c is downregulated in prostate cancer and suppresses prostate cancer growth. PLoS ONE.

[20] Kong D., Heath E., Chen W., Cher M.L., Powell I., Heilbrun L., Li Y., Ali S., Sethi S., Hassan O. (2012). Loss of Let-7 upregulates EZH2 in prostate cancer consistent with the acquisition of cancer stem cell signatures that are attenuated by BR-DIM. PLoS ONE.

[21] Muraca M., Cappariello A. (2020). The role of Extracellular Vesicles (EVs) in the epigenetic regulation of bone metabolism and osteoporosis. Int. J. Mol. Sci..

[22] Wang Z., Xu L., Hu Y., Huang Y., Zhang Y., Zheng X., Wang S., Wang Y., Yu Y., Zhang M. (2016). miRNA Let-7b modulates macrophage polarization and enhances tumor-associated macrophages to promote angiogenesis and mobility in prostate cancer. Sci. Rep..

[23] Bellezza I., Aisa M.C., Palazzo R., Costanzi E., Mearini E., Minelli A. (2005). Extracellular matrix degrading enzymes at the prostasome surface. Prostate Cancer Prostatic Dis..

[24] Burden H.P., Holmes C.H., Persad R., Whittington K. (2006). Prostasomes--Their effects on human male reproduction and fertility. Hum. Reprod. Update.

[25] Gregory C.D., Paterson M. (2018). An apoptosis-driven ‘onco-regenerative niche’: Roles of tumour-associated macrophages and extracellular vesicles. Philos. Trans. R. Soc. Lond. B. Biol. Sci..

[26] Lin F., Yin H.B., Li X.Y., Zhu G.M., He W.Y., Gou X. (2020). Bladder cancer cell-secreted exosomal miR-21 activates the PI3K/AKT pathway in macrophages to promote cancer progression. Int. J. Oncol..

[27] Valadi H., Ekström K., Bossios A., Sjöstrand M., Lee J.J., Lötvall J.O. (2007). Exosome-mediated transfer of mRNAs and microRNAs is a novel mechanism of genetic exchange between cells. Nat. Cell Biol..

[28] Banerjee S., Xie N., Cui H., Tan Z., Yang S., Icyuz M., Abraham E., Liu G. (2013). MicroRNA Let-7c regulates macrophage polarization. J. Immunol..

[29] Coley W., Van Duyne R., Carpio L., Guendel I., Kehn-Hall K., Chevalier S., Narayanan A., Luu T., Lee N., Klase Z. (2010). Absence of DICER in monocytes and its regulation by HIV-1. J. Biol. Chem..

[30] Wang J., Ni J.M., Beretov J., Thompson J., Graham P., Li Y. (2020). Exosomal microRNAs as liquid biopsy biomarkers in prostate cancer. Crit. Rev. Oncol. Hematol..

[31] Chirshev E., Oberg K.C., Ioffe Y.J., Unternaehrer J.J. (2019). Let-7 as biomarker, prognostic indicator, and therapy for precision medicine in cancer. Clin. Transl. Med..

[32] Urbanelli L., Buratta S., Sagini K., Ferrara G., Lanni M., Emiliani C. (2015). Exosome-based strategies for Diagnosis and Therapy. Recent Pat. CNS Drug Discov..

